# Serological positive markers of hepatitis B virus in femoral venous blood or umbilical cord blood should not be evidence of in-utero infection among neonates

**DOI:** 10.1186/s12879-016-1754-1

**Published:** 2016-08-12

**Authors:** Lei Zhang, Xi-En Gui, Bo Wang, Jing-Yi Fan, Qian Cao, Kathleen Mullane, Xiao-Li Liang

**Affiliations:** 1 Department of Infectious Diseases, Zhongnan Hospital of Wuhan University, No.169, Donghu Road, Wuchang District, Wuhan City, Hubei Province, People’s Republic of China; 2 Department of Infection Control, Qingdao Municipal Hospital, Qingdao, China; 3 Department of gynaecology and obstetrics, Infectious Disease Hospital, Taiyuan, China; 4 Department of Paediatrics, Zhongnan Hospital of Wuhan University, Wuhan, China; 5 Department of Medicine/Division of Infectious Diseases, University of Chicago, Chicago, USA

**Keywords:** HBV, Serological markers, Maternal-infant transmission, in-utero, Delivery, Immunoprophylaxis failure

## Abstract

**Background:**

Maternal-infant transmission of hepatitis B virus(HBV) occurs even after passive-active immunization. Some scholars speculate that in-utero infection may be the main cause of immunoprophylaxis failure. However, there is a lack of evidence about the possible occurrence periods of perinatal transmission.

**Methods:**

From 2008 to 2012, 428 pairs of HBsAg-positive mothers and neonates were enrolled and 385 infants aged 8–12 months were followed. HBV markers (HBsAg, anti-HBs, HBeAg, anti-HBe, anti-HBc, HBV-DNA) were performed on all subjects.

**Results:**

Of mothers who were positive for HBsAg, HBeAg, HBV-DNA, 35.1 %, 94.3 %, 12.7 % of their neonates were positive for those indices, respectively. Neonates’ mean titers of those indices were significantly lower than their mothers’. There were no significant differences in rates of positivity and mean titers of anti-HBe and anti-HBc between neonates and mothers. Most of the positive indices turned negative during the follow-up period. Immunoprophylaxis failed in seventeen infants: four infants had HBV-DNA > 6 log _10_copies/mL both at birth and in follow-up; in six infants, mean viral load was 3.72 ± 0.17 log _10_copies/mLat birth and 7.62 ± 0.14 log _10_copies/mL at follow-up; seven infants were HBV-DNA negative at birth but were found to have > 6 log _10_copies/mL during follow-up. Infants that were immunoprophylaxis failures were all born to HBeAg-positive mothers with HBV-DNA > 6 log _10_copies/mL.

**Conclusions:**

The placental barrier can partly prevent maternal HBsAg, HBeAg, HBV-DNA from passing through to fetus. Performing HBsAg, HBeAg, HBV-DNA once at birth can neither diagnose nor exclude maternal-infant transmission. The diagnosis of infection period depends on the dynamic changes in viral load from birth through the follow-up period but whether the infection occurred in utero, at delivery or during the neonatal period could not be determined.

## Background

Hepatitis B virus (HBV) infection is a major public health problem in the world with about 2 billion people who have been infected with HBV [1]. The World Health Organization reported that there are an estimated 240 million chronically infected persons worldwide, particularly in low-and middle-income countries, and an estimated 650,000 people die annually due to the major complications of chronic hepatitis B, cirrhosis and hepatocellular carcinoma [2]. China is a high prevalence area, with HBsAg seropositivity in the population reported as 9.8 in 1992 and 7.2 % in 2006 [3]. Maternal-infant transmission is the major route for HBV transmission and subsequent chronic infectivity, accounting for up to 30 % of cases [4]. Hepatitis B vaccination of newborn infants reduces the likelihood of perinatal transmission from HBeAg-positive mothers by 79–90 %, and the likelihood is further reduced by adding concurrent administration of hepatitis B immunoglobulin (HBIG), a regimen (passive-active immunoprophylaxis) that is 85–95 % effective in preventing development of a chronic HBV carrierstate [5–7].

Before the initiation of vaccination programs for newborns, three modes of transmission of HBV from carrier mothers to infants were considered possible: in utero, during delivery and postnatally [8, 9]. After the implementation of passive-active immunoprophylaxis, the small proportion of failures are presumed to be in those infants who are infected in utero and already have an established infection at birth [10, 11]. These factors have led clinicians in China to conclude that the appearance of those indices in newborns is an indicator of intrauterine infection [12–14]. However, there is little evidence to determine whether or not in-utero transmission of HBV is the major route of perinatal transmission when infants have received passive-active immunoprophylaxis or if other possible routes of perinatal infection are more common than previously believed.

Serological characteristics of hepatitis B markers in neonate were used to analysis the periods of HBV maternal-infant transmission by some scholars in China. Umbilical cord (UC) blood drawing at delivery is comperatively easy, timely, safe and acceptable by parents. Some scholars considered that umbilical cord blood might be contaminated by maternal blood, which may lead to misleading results, and so they prefer to femoral venous (FV) blood.

This study aimed to explore the possible occurrence periods of HBV maternal-infant transmission by analyzing the serological characteristics of hepatitis B markers between HBsAg-carrier mothers and their infants. Further, both the UC and FV were collected and compared in this study.

## Methods

### Study population and sites

From January 2008 to December 2012, this prospective study was conducted in Zhongnan Hospital of Wuhan University and its peripheral hospitals (Mother and Child hospitals of Dangyang city, Tongcheng county, Huanggang city and Xiaonan district of Xiaogan city) in Hubei province and in the Infectious Disease Hospital of Taiyuan City in Shanxi province. HBsAg-carrier mothers and their neonates were enrolled in this study; follow-up was done when infants reached 8–12 months of age. HBV markers (HBsAg, anti-HBs, HBeAg, anti-HBe, anti-HBc and HBV-DNA) were measured for all mothers, neonates and during follow-up of these infants.

Women with more than twice the upper limit of normal for alanine aminotransferase or other complications of gestation were excluded from this study. Women involved in the study were not vaccinated against HBV.

All normal births were included in this study. Neonates that were preterm, weighed 2500 g or less, or had an Apgar score <8 were excluded from this study. Caesarian sections were not a factor in inclusion or exclusion criteria.

### Sample collection

One staff member from each participating center was trained in completion of a unified questionnaire for each mother-infant pair, coordinating follow-up visits and handling bio hazardous clinical samples.

Venous blood samples were obtained from pregnant women during their second or third trimester and from infants at birth and follow-up visits. Blood was collected from neonates at the Infectious Disease Hospital of Taiyuan City via FV blood prior to immunization while neonates in Zhongnan Hospital of Wuhan University and its peripheral hospitals in Hubei province (Mother and Child hospitals of Dangyang city, Tongcheng county, Huanggang city and Xiaonan district of Xiaogan city) had venous samples collected via UC blood prior to immunization. The volume of blood drawn was 2–3 ml for each subject. The serum was separated within 1 h, transferred into a 1.5 ml centrifuge tube and stored in a – 70 °C refrigerator for HBV marker testing.

### HBV immunoprophylaxis for infants

Passive and active immunization was given to neonates born to HBsAg-carrier mothers. Within 24 h of delivery. Each neonate was given 100 IU HBIG (Rongsheng Pharmacy Company of Chengdu, Sichuan Province, China) and 10 μg Hepatitis B vaccine (Yeast recombinant hepatitis B vaccine of Beijing Tiantan Biological Products Co.,LTD) by intramuscular injection. Vaccination was repeated with the same dosage at 1 and 6 months of age.

### Diagnosis of immunoprophylaxis failure

Infants aged 8–12 months with negative HBsAg, HBeAg and HBV-DNA were considered HBV uninfected. Infants with positive HBsAg, HBeAg and HBV-DNA were considered HBV infected and were considered as an immunoprophylaxis failure.

### Laboratory methods

HBsAg, anti-HBs, HBeAg, anti-HBe and anti-HBc were performed by Cobas e601 analyzer, Roche, Germany in those specimens drawn via the femoral vein while those drawn from the umbilical vein were performed by Cobas e411 analyzer, Roche, Germany. The normal reference values for both analyzers were: HBsAg < 1.00 Cut-off-index (COI), anti-HBs 0–10 IU/L, HBeAg < 1.00 COI, anti-HBe > 1.00 COI, anti-HBc > 1.00 COI (The anti-HBc was total core). The results of HBsAg, HBeAg, HBeAb and HBcAb are semi-quantitative assessments and the result of anti-HBs is quantitative assessment.

The data in this study was obtained from two provinces, Hubei province (central China) and Shanxi province (northern China). To avoid detection error caused by impaired specimen quality due to long-distance transportation, the testing was performed in the two centers respectively. Samples of UC blood were obtained in Hubei province (in Wuhan and its peripheral cities) and were analyzed in Zhongnan Hospital of Wuhan University where the instrument Cobas e411 was available. Samples of FV blood were obtained in Shanxi province (the Infectious Disease Hospital of Taiyuan City) and were analyzed in the Infectious Disease Hospital of Taiyuan City where the instrument Cobas e601 was available. The two instruments were both produced by Roche Diagnostics Company in Germany and were utilized with the same testing principles.

The instrument, Cobase411 or 601, is a full-automatic electro chemiluminescence immunoassay system. The accuracy, precision, sensitivity and specificity are high, and repetitive testing is not needed.

HBV-DNA was performed by using PCR-Fluorescence detection kits for hepatitis B viral nucleotides (Shanghai Kehua Bio-engineering Co., Ltd, China). Reference to the instruction of the test kit, the positive level was set as HBV-DNA ≥ 500 copies/ml.

### Statistical analysis

The normal distribution of measurement data was first tested. If data was of non-normal distribution, nonparametric tests were used for statistical calculations, expressed in terms of the median (25 % to 75 % inter quartile range, IQR). If the data was of normal distribution,t tests or nonparametric tests were used, expressed as $\overline{x}\pm$ SD or median (25 % to 75 %IQR). SPSS 17.0 software package was used for analysis, and a *P* value of < 0.05 was considered significant.

## Results

### Study population

Among samples meeting the requirements, 428 (FV, 301; UC, 127) matched maternal-neonatal blood samples were collected and 385 (90.0 %) (FV, 271; UC, 114) infants were included in follow-up at the age of 8–12 months. Forty-three infants were lost in follow-up because they left their city of residence after delivery or because their parents refused to have the child’s blood drawn. There were no significant differences between the successful and unsuccessful follow-up groups with regard to positive rates of HBV markers of mothers and their infants (Table 1).

**Table 1:** HBV markers in groups of follow-up and lost to follow-up

Items	Markers	Follow-up	Lost to follow-up	*P*-value
Mothers	HBsAg(+)	100.0 % (385/385)	100.0 % (43/43)	/
	HBeAg(+)	50.1 % (193/385)	44.2 % (19/43)	0.460
	HBV-DNA(+)	57.1 % (220/385)	53.5 % (23/43)	0.646
	anti-HBe(+)	47.0 % (181/385)	41.9 % (18/43)	0.521
	anti-HBc(+)	100.0 % (385/385)	100.0 % (43/43)	/
Infants	HBsAg(+)	35.1 % (135/385)	41.9 % (18/43)	0.378
	HBeAg(+)	47.3 % (182/385)	44.2 % (19/43)	0.700
	HBV-DNA(+)	7.3 % (28/385)	14.0 % (6/43)	0.215
	anti-HBe(+)	44.9 % (173/385)	39.5 % (17/43)	0.499
	anti-HBc(+)	99.0 % (381/385)	100.0 % (43/43)	1.000

### Semi-quantitative estimation of HBV markers in HBsAg-carrier mothers and their neonates

In the 385 neonates of HBsAg-positive mothers, they were collected by either FV blood or UC blood prior to immunization and the infants were later available for follow-up at the age of 8–12 months. Results suggest that HBsAg, HBeAg and HBV-DNA could be partially prevented from passing through the placenta but anti-HBe and anti-HBc were frequently passed to the fetus.

Ratios of positive HBV markers, trans placental markers, in neonates compared with their HBsAg-carrier mothers were HBsAg, 35.1 (135/385), HBeAg, 94.3 (182/193), HBV-DNA, 12.7 (28/220), anti-HBe, 95.6 (173/181) and anti-HBc, 99.0 % (381/385). Neonates positive for HBeAg were all born to HBeAg-positive mothers. The ratio for each marker was similar in both the FV and UC groups (Table 2). Semi-quantitative values of HBV markers in matched mother-neonate pairs are reported in Table 3.

**Table 2:** The counts of positive HBV markers of neonates comparing with those of mothers

Items	Source	Mother(M)	Neonate (N)	N/M	*P*-value
		No. (+)	No. (+)	%	
HBsAg	FV	271	87	32.1	0.060
	UC	114	48	42.1	
HBeAg	FV	141	136	96.5	0.076
	UC	52	46	88.5	
HBV-DNA	FV	161	18	11.2	0.255
	UC	59	10	16.9	
anti-HBe	FV	122	118	96.7	0.491
	UC	59	55	93.2	
anti-HBc	FV	271	269	99.3	0.728
	UC	114	112	98.2

**Table 3:** Quantitative values of HBV markers in matched mothers and neonates

Items		n	Mother (M)	Neonate (N)	*P*-value	N/M (Median)
			Median (25 %, 75 % IQR)	Median (25 %, 75 % IQR)		
HBsAg(+)	FV	87	795.30 (340.28, 2341.25)	1.96 (1.19, 4.56)	0.000	0.002
	UC	48	2834.50 (1557.75, 7907.25)	3.84 (2.15, 36.80)	0.000	0.001
HBeAg(+)	FV	136	588.65 (282.33, 790.35)	41.45 (12.90, 128.71)	0.000	0.070
	UC	46	1140.50 (579.08, 1536.25)	49.38 (11.83, 317.65)	0.000	0.043
HBV-DNA(+)	FV	18	7.10 (6.88, 8.02)	3.0 (3.0, 3.48)	0.000	0.0001
	UC	10	7.59 (6.50, 7.74)	3.84 (3.52, 4.94)	0.004	0.00018
anti-HBe(+)	FV	118	0.029 (0.005, 0.069)	0.012 (0.004, 0.058)	0.073	/
	UC	55	0.004 (0.003, 0.010)	0.003 (0.002, 0.010)	0.428	/
anti-HBc(+)	FV	269	0.006 (0.004, 0.080)	0.006 (0.004, 0.072)	0.688	/
	UC	112	0.006 (0.005, 0.007)	0.006 (0.005, 0.006)	0.144	/

HBsAg (COI), HBeAg (COI), HBVDNA( log _10_copies/mL), anti-HBe (COI), anti-HBc (COI); /:mean titer was not compared between neonates and mothers because the index was measured by competitive binding principle

All the mothers and neonates at the time of birth were negative for anti-HBs.

### Changes in HBV markers during follow-up

**Fig. 1:**
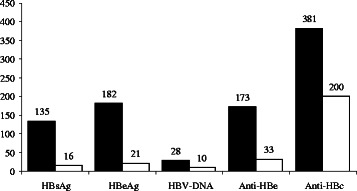
The loss rates of HBV markers in follow-up infants. Black bars represent at birth and white bars represent at follow-up. The loss rates of HBV markers were: HBsAg, 88.1 %, HBeAg, 88.5 %, HBV-DNA, 64.3 %, anti-HBe, 80.9 %, anti-HBc, 47.5 %

Three hundred and eighty-five infants were followed up for testing of HBV markers at 8–12 months of age (Fig. 1). Serologic markers most likely to be lost were, HBeAg and HBsAg followed by HBV-DNA, anti-HBe and anti-HBc, respectively.

The loss of HBV markers among infants during follow-up was similar in both the FV and UC groups (Table 4). Anti-HBs were not found in any neonates at birth. After completion of 3-dose hepatitis B vaccine series, 354 out of 385 (91.9 %) infants were positive for anti-HBs (>10 IU/L). Among 368 babies without HBV infection, 16 had inadequate anti-HBs (<10 IU/L). No HBeAg-anti-HBe seroconversion was found.

**Table 4:** Loss of HBV markers during follow-up among infants in FV group and UC group

Items	Source	At birth	8–12 months	Loss of HBV markers	*P*-value
		n (+)	n (+)	%	
HBsAg	FV	87	10	88.5	0.863
	UC	48	6	87.5	
HBeAg	FV	136	13	90.4	0.151
	UC	46	8	82.6	
HBV-DNA	FV	18	6	66.7	1.000
	UC	10	4	60.0	
anti-HBe	FV	118	20	83.1	0.297
	UC	55	13	76.4	
anti-HBc	FV	269	134	50.2	0.105
	UC	112	66	41.1	

One hundred and thirty-five out of 385 neonates were positive for HBsAg. Among those, 98 were HBeAg positive, and 28 were positive for both HBV-DNA and HBeAg at birth. Ten of the 28 had immunoprohylaxis failure during follow-up; five with HBsAg positive, HBeAg positive and HBV-DNA negative at birth had immunoprohylaxis failure; one with HBsAg positive, HBeAg negative and HBV-DNA negative at birth had immunoprohylaxis failure during follow-up.

Among the 135 neonates, there was no one with HBV-DNA positive but HBeAg negative at birth.

Two hundred and fifty out of 385 infants were negative for HBsAg. Among those, 84 were HBsAg negative, HBeAg positive and HBV-DNA negative at birth, and one of the 84 had immunoprohylaxis failure during follow-up (Table 5); 165 were negative for HBsAg, HBeAg and HBV-DNA both at birth and during follow-up.

**Table 5:** Serum testing results in immunoprophylaxis failure infants and their mothers

Group	No.	Mother			Infant					
					At birth			8–12 months		
		HBsAg	HBeAg	HBV-DNA	HBsAg	HBeAg	HBV-DNA	HBsAg	HBeAg	HBV-DNA
1	a	2685	1024	8.2	8537	897.9	6.2	968.2	1626	8.7
	b	364.5	729.8	8.1	560.3	44.74	6.5	360.5	1516	7.4
	c*	3319	447.9	8.2	6205	6.76	6.5	1647	1110	7.9
	d*	1719	1762	7.6	1244	1771	8.5	901.1	1898	8.6
2	a	286.5	1367	6.5	122.3	22.08	3.8	256	900	7.4
	b	98.13	305.6	7.6	6.74	138.2	3.9	391.1	878.1	8.0
	c	1318.4	221.3	7.2	3.92	25.24	3.3	581.6	1220	7.1
	d	47.8	27.6	6.5	5.13	9.02	4.3	2380	885.2	7.7
	e*	1284	1226	7.6	3.71	11.51	3.8	980.3	1279	8.0
	f*	3350	5020	7.7	55.72	+	3.2	2466	1256	7.5
3	a	379.6	961.4	6.9	2.97	12.25	—	378.8	565.1	7.6
	b	971.6	9.707	7.4	4616	106.4	—	220	244.1	6.2
	c	478.7	829.2	6.6	26.06	10.02	—	349.6	18.3	8.3
	d	1419	813.9	6.8	1.39	108.2	—	3732	907.3	6.5
	e	3255	785.1	6.6	0.48	11.44	—	4681	960.4	6.8
	f*	1975	1316	7.3	17.66	+	—	5967	1449	5.7
	g*	2407	1299	7.9	1.0	14.09	—	25.03	897.1	4.6

The number labeled “*” represents UC and without “*” represents FV, HBsAg (COI), HBeAg (COI), HBV-DNA (log _10_copies/mL); —: HBV-DNA < 500 copies/mL;+: This index was positive for qualitative analysis according to previous assay sheet and not tested by quantitative analysis because of insufficient sample

Among infants followed-up, there was no discordance in markers e.g. HBeAg and/or HBV-DNA positive but HBsAg negative.

### Cases of immunoprophylaxis failure

Seventeen of the 385 infants born to HBsAg positive mothers were HBsAg, HBeAg and HBV-DNA positive at age 8–12 months (Table 5). These infants were considered to be immunoprophylaxis failures, accounting for 4.4 % (17/385) of all infants who received passive-active immunization. These 17 infants were all born to HBeAg positive mothers whose HBV-DNA were > 6 log _10_copies/mL.

The immunoprophylaxis failure rate was 8.8 % (17/193) in the group of HBeAg-positive mothers. The failure rates were 0 (0/164), 0(0/53), 9.7 (6/62), 10.3 (8/78) and 10.7 % (3/28) in those infants born to subjects with maternal HBV-DNA < 500 copies/ml, 2–5.99 log_10_copies/ml, 6–6.99 log_10_copies/ml, 7–7.99 log_10_copies/ml and ≥8 log _10_copies/ml, respectively.

Maternal HBsAg load, indicated with median (25 %,75 % IQR), in the group of infants with HBV infection and 368 infants without HBV infection were 1351.5 (368.3, 2615.5) COI and 1235.0 (162.1, 3983.0) COI. Compared both, *p* = 0.747.

The 17 infants were divided into 3 groups, group 1 (a-d), group 2 (a-f), group 3 (a-g), based on their levels of HBV-DNA viral load at birth (Table 5).

Twenty-eight infants were positive for HBV-DNA, HBsAg and HBeAg at birth and 18 (64.3 %) seroconvert during the follow-up period. The mean level of HBV-DNA was 3.48 ± 0.68 log _10_copies/mL for these 18 infants at birth. This indicates that these infants were exposed to HBV in utero or during delivery and were protected by passive-active immunization.

HBV-DNA in four cases remained positive and above 6 log _10_copies/mL. The mean viral load was 6.93 ± 0.53 log _10_copies/mL at birth and 8.15 ± 0.31 log _10_copies/mL at follow-up (t = −2.001, *p* = 0.092).

HBV-DNA levels of another six infants rose from a lower level at birth to a higher level during follow-up. In these infants, the mean viral load was 3.72 ± 0.17 log _10_copies/mL at birth and 7.62 ± 0.14 log _10_copies/mL in follow-up (t = −17.699, *p* = 0.000).

Seven infants were negative for HBV-DNA at birth but became positive in follow-up period with mean viral load of 6.43 ± 1.15 log _10_copies/mL. Six of these infants were formula-fed and one was breast-fed after birth.

Anti-HBs antibodies were negative for those who had immunoprophylaxis failure.

## Discussion

Institution of nationwide HBV immunization has successfully reduced the seroprevalence of HBV in China. The seroprevalence of HBsAg has decreased markedly from over 8.5 % in 1992 to less than 1.0 % in 2006 among children between the ages of one to 4 years since the Chinese CDC recommended routine immunization with hepatitis B vaccine [15]. Despite the implementation of immunization measures, the maternal-infant transmission rate of HBV was 4.4 % among all infants in this study. The immunoprophylaxis failure rate was noted in this study to be higher than previously reported in published studies [16–18]. The infants considered as immunoprophylaxis failures in this prospective analysis were all born to HBeAg positive mothers whose HBV-DNA were >6 log _10_copies/mL. The maternal-infant transmission rate was 8.8 % in the group of HBeAg-positive mothers. Therefore, in this patient population, mothers who had high levels of viremia formed the primary grouping which HBV maternal-infant transmission occurred and would therefore likely be a key population to target in an effort to control HBV perinatal transmission.

Epidemiological evidence has proven the existence of HBV maternal-infant transmission but the exact mode of transmission of infection is unclear. Due to the development and the availability of advanced measuring instruments, we had the ability to conduct this study with a higher measure of accuracy than was previously available in China.

It has been suggested that a fetus may obtain maternal HBV markers passed through placenta [19–22]. In this study, we determined the extent to which these HBV markers can be passed transplacentally in clinical settings. In this analysis, HBsAg positive rate of neonates was 35.1% and neonatal titers of HBsAg were significantly lower than those of the infants’ mothers. This finding suggests that the majority of HBsAg, in indices by either the transplacental rate or the amount, were not passed through the placental barrier. Transplacental transfer of HBeAg occurred in 94.3 % of cases, but the concentration in neonates was 4.3–7.0 % of maternal levels. There was little transplacental HBV-DNA transfer, with the titer in neonates being approximately 0.01–0.018 % of their mothers. The transplacental rate of anti-HBe transference was 95.6 and 99.0 % for anti-HBc, with no significant difference in median titers between neonates and their mothers. These results indicate that the placental barrier can prevent maternal-infant transmission of HBsAg, HBeAg and HBV-DNA, but it transfers the antibodies, anti-HBe and anti-HBc, from mother to fetus in the vast majority of cases. This can be explained by the fact that maternal IgG antibodies are actively transferred through the placenta.

The capability of HBsAg, HBeAg and HBV-DNA to pass through the placental barrier may depend upon their molecular sizes or differences in HBV particle morphologies [23, 24]. HBeAg is a soluble antigen with a small molecular weight, and it easily passes through the placenta. HBsAg, expressed on spherical or filamentous particles as well as Dane’s particles, passes through the placental barrier with relative difficulty compared to HBeAg. HBV-DNA is enveloped in a Dane particle, which is frequently prevented from passing across the placental barrier.

In this study, the methodology of the testing reagents for anti-HBe and anti-HBc and the analytical instrumentation were designed by the competitive binding principle, meaning a lower value indicates a higher concentration of antibody present. The median titers of neonates for anti-HBe and anti-HBc were higher than or equal to those of mothers, respectively (Table 3). Our results mirror a similar report [25], which is because placental transfer of IgG is an active process by means of the binding of maternal IgG to neonatal Fc receptors in the placenta [26].

Our present study further showed that in infants, approximately 88.1 HBsAg, 88.5 HBeAg and 64.3 % HBV-DNA disappeared at the age of 8–12 months. This may simply represent transplacental maternal (HBsAg and HBeAg) non-infectious antigens rather than infectious HBV particles replicating in the infants’ own bodies. Alternatively, the immunoprophylaxis measures (HBIG plus hepatitis B vaccine) administered to neonates, especially for those with low titer of HBV-DNA at birth, may prevent part of the maternal-fetal transmission that occurs during delivery. The appearance of these antigens at birth may not be an indication that neonates are infected with HBV in-utero as infection in-utero may lead to fetal immuno tolerance to HBV and a chronic hepatitis B carrying status [27, 28].

Our results show transplacental anti-HBe disappeared in 80.9 % infants before 8-12 month of age, and new anti-HBe antibodies were not produced in the immunoprophylaxis protected infants born to HBeAg-positive mothers. In view of these findings, in-utero infection may occur less frequently than previously speculated. Transplacental anti-HBc can last a longer period of time than anti-HBe in infants born to HBsAg-carriermothers. Anti-HB was detected in about half of the infants in this study at the age of 8–12 months. And it had previously been reported to disappear before 24 months of age [29]. Therefore, the sole presence of anti-HBe before 12 months of age with or without anti-HBc before 24 month of age in immunoprophylaxis protected infants born to HBsAg-carriermothers suggests the transplacental transfer of maternal antibodies to the infants, and may not indicate that the infants have experienced viral infection in-utero.

Thus, these positive indices at birth that disappear during follow-up are transferred from the mothers, rather than through HBV replication in the infants. This is an important difference between infants with positive HBV markers and adults who suffer from HBV infection. Previously published opinions which state that HBsAg and/or HBV-DNA positivity in neonates at birth should be criteria for intrauterine infection [12–14] may have failed to consider the fact that loss of HBV markers in follow-up is inconsistent with intrauterine infection. Our finding is consistent with a study by Papaevangelou V, et al. [30], which reaches a similar conclusion that the presence of HBV-DNA in newborns may not represent HBV infection.

Among the seventeen infants of immunoprophylaxis failure, four may represent intrauterine infection, because in those infants HBV-DNA viral load was > 6 log _10_copies/mL both at birth and at 8–12 months of age. The results suggest that the neonates were likely to be infected with HBV in utero, but this study cannot demonstrate that infection definitely occurred in-utero, because some newborns maybe exposed to large amount of HBV during delivery, leading to high levels of HBV-DNA at birth [31].

In six other infants, mean viral-loads of HBV-DNA were significantly lower at birth than were their follow-up viral loads. The HBV-DNA level of these six infants (3.72 ± 0.17 log _10_copies/mL) was similar to that of the eighteen infants (3.48 ± 0.68 log _10_copies/mL) whose HBV-DNA was positive at birth and negative at follow-up visits. Since those eighteen infants were able to clear the virus after immunoprophylaxis, the six infants with low level of HBV-DNA at birth might have been HBV positive at birth with immunoprophylaxis failure. However, we cannot completely rule out the possibility of these infants being infected in the late stage of pregnancy.

Notably, there were seven neonates that tested HBV-DNA negative at birth but found to be HBV-DNA positive at 8–12 months of age, with six of them being formula feed. For these six infants, transmission during breastfeeding can be excluded. If an infant has received immunoprophylaxis, infection through breast milk is not considered as a factor to put the child at risk of maternal-infant transmission [18, 32]. Therefore, the findings suggest these infants were more likely to have been infected during delivery, but in-utero infection cannot be excluded when HBV markers are negative at birth because in-utero infection that occur 1–2 weeks antepartum may have negative HBV markers due to the relatively long period of incubation time of HBV infection.

In this study, 165 (42.9 %) were negative for HBsAg, HBeAg and HBV-DNA both at birth and during follow-up. These infants were followed at 8–12 months of age after birth, when they have had a complete course of vaccination and infants’ daily activities were mainly happened with their mothers, that is, there is almost no chance of other HBV exposure. So apart from “accidental infection”, the possibility of inadequate immune response and postpartum infection can be excluded. This suggests that these infants did not become HBV infected *in utero* or during delivery and remained infection-free at follow-up, although it cannot be ascertained that immunoprophylaxis was effective.

HBV markers, HBsAg, HBeAg and/or HBV-DNA, in high risk infants at birth, disappeared at 8-12 months with the loss rate of 88.1 %, 88.7 %, 64.3 %, respectively. Conversely, these markers, negative at birth, became positive in a small number of infants during the follow-up period. Therefore, positivity of HBV markers at birth cannot universally be used to define in-utero or maternal-infant transmission, and negativity for HBV markers cannot be used to exclude maternal-infant transmission. The dynamic changes of viral load in HBV infected infants may indicate the estimated possible infection period (Table 6), but it is still impossible to definitively differentiate in-utero infection from delivery infection. Therefore, monitoring high-risk infants during follow-up is essential for the determination of maternal-infant transmission. We suggest that follow-up studies should be done at 8–12 months age because the markers of both infection (HBsAg) and immunity (anti-HBs) may develop after immunization in this high risk population.

**Table 6:** Tendency of infection periods among infants of immunoprophylaxis failure

Group	HBV-DNA viral load		Intrauterine infection	Delivery infection
	At birth	Follow up		
1(a-d)	high^a^	high	+++	+
2(a-f)	low^b^	high	++	++
3(a-g)	negative	high	+	+++

More “+” means the degree of possibility is more likely

^a^HBV-DNA > 6 log _10_copies/mL

^b^HBV-DNA <4 log _10_copies/mL

In this study, whether or not the specimens were collected from neonates via the FV or the UC, a similartransplacental rate of acquisition and loss of each HBV marker was noted both at birth and during the follow-up period. The subgroup analysis confirmed the results’ reliability. The results also indicate that both UC and FV blood drawing share the same feasibility. So, compared with FV, UC drawing might be a better way of blood drawing for neonates by its characteristics of comparatively easy, timely, safe and acceptable by parents.

Passive-active prophylaxis has been demonstrated to be effective in preventing HBV infection in most of infants born toHBsAg-carrier mothers. However, HBV maternal-infant transmission still occurs after passive-active immunization. This study suggests that the majority infants with immunoprophylaxis failure were infected with HBV during delivery. It may be more effective to give neonates a higher titer of HBIG in reducing the rate of perinatal transmission. It is reported that the efficacy of monoclonal antibody of HBIG was 100 times the titer of HBIG available now with the same dosage [33]. It may be worthwhile to try this antibody in preventing maternal-infant transmission, but this work needs further investigation.

It’s also possible that a small part of immunoprophylaxis failure in infants occurred due to in-utero infection. Administration of antiviral therapy to lower the maternal serum HBV-DNA levels may reduce the rate of intrauterine infection. Preliminary studies have suggested that nucleoside analogues against HBV infection, (lamivudine or telbivudine) may reduce HBV intrauterine infection [34, 35], but there is still not enough evidence regarding the safety and effectiveness of this treatment in pregnant women, especially for symptom-free hepatitis B carriers. Large clinical trials are needed to further investigate the efficacy of anti-HBV therapy for pregnant women in preventing HBV maternal-infant transmission in pregnant women.

Study limitations: First, there was only one follow-up time point, from which it is impossible to estimate either when HBV markers became undetectable or when these markers became detectable. Further studies are needed to explore the exact modes of HBV maternal-infant transmission, which will inform the implementation of more effective measures to prevent perinatal infection. Second, this study did not perform molecular characterization of the HBV isolated from the mothers compared to the isolates from the neonates. Differing HBV viral strains may have been a confounding factor in the vertical transmission and immunoprophylaxis failure together with or irrespective of viral load. This is also a subject of further study.

## Conclusions

This prospective study described HBV maternal-infant transmission in a high risk group of maternal-infant pairs in China and analyzed the possible periods of transmission based on serological characteristics of matched maternal-infant samples. HBV maternal-infant transmission still occurred after passive-active immunization. This study provided evidence that the placental barrier can partly prevent maternal HBV markers from passing through to fetus; that fetuses got their maternal HBV markers do not represent true infection of HBV; the dynamic changes in viral load from birth through the follow-up period could be referred to for the diagnosis of infection period; in-utero infection may occur less frequently than previously speculated. Above all, although even today, with sensitive testing, the exact mode of maternal-infant transmission, in utero, at delivery or during the neonatal period, could not be exactly differentiated, but what seems certain is that serological positive markers of HBVin femoral vein blood or umbilical cord blood should not be evidence of in-utero infection among neonates.

## Abbreviations

FV, femoral venous; HBIG, hepatitis B immunoglobulin; HBV, hepatitis B virus; IQR, inter quartile range; UC, umbilical cord.
